# Regulation of alternative splicing in obesity-induced hypertension

**DOI:** 10.2147/DMSO.S188680

**Published:** 2019-08-28

**Authors:** Zodwa Dlamini, Rodney Hull, Tshepiso J Makhafola, Mzwandile Mbele

**Affiliations:** 1 South African Medical Research Council/University of Pretoria Precision Prevention & Novel Drug Targets for HIV-Associated Cancers (PPNDTHAC) Extramural Unit, Pan African Cancer Research Institute (PACRI), Faculty of Health Sciences, University of Pretoria, Hatfield 0028, South Africa

**Keywords:** obesity, hypertension, weight loss, angiogenesis, alternative splicing, gene regulation

## Abstract

Obesity is the result of genetics which predisposes an individual to obesity and environmental factors, resulting in excessive weight gain. A well-established linear relationship exists between hypertension and obesity. The combined burden of hypertension and obesity poses significant health and economic challenges. Many environmental factors and genetic traits interact to contribute to obesity-linked hypertension. These include excess sodium re-absorption or secretion by the kidneys, a hypertensive shift of renal-pressure and activation of the sympathetic nervous system. Most individuals suffering from hypertension need drugs in order to treat their raised blood pressure, and while a number of antihypertensive therapeutic agents are currently available, 50% of cases remain uncontrolled. In order to develop new and effective therapeutic agents combating obesity-induced hypertension, a thorough understanding of the molecular events leading to adipogenesis is critical. With the advent of whole genome and exome sequencing techniques, new genes and variants which can be used as markers for obesity and hypertension are being identified. This review examines the role played by alternative splicing (AS) as a contributing factor to the metabolic regulation of obesity-induced hypertension. Splicing mutations constitute at least 14% of the disease-causing mutations, thus implicating polymorphisms that effect splicing as indicators of disease susceptibility. The unique transcripts resulting from the alternate splicing of mRNA encoding proteins that play a key role in contributing to obesity would be vital to gain a proper understanding of the genetic causes of obesity. A greater knowledge of the genetic basis for obesity-linked hypertension will assist in the development of appropriate diagnostic tests as well as the identification of new personalized therapeutic targets against obesity-induced hypertension.

## Introduction

Obesity is a health problem, affecting individuals across the globe, and is on the increase in both emerging countries and the western world, regardless of gender race, ethnicity or socio-economic status. Recent estimates place the number of overweight adults at 1.9 billion worldwide, with 650 million being obese.^1^ Obesity is reported to reduce the life expectancy of adults by 4 to 10 years and 3 million deaths are attributed to severe cases of obesity annually.^2^ Another critical concern is that in 2016 it was estimated that 40 million children under the age of 5 were obese and this number is increasing.^2^ The epidemic of childhood obesity is expected to cause a surge in cardiovascular diseases. Obesity also predisposes individuals to hypertension, diabetes, cancer, and musculoskeletal disorders.^1^,^3^–^5^ Both high blood pressure and diabetes are most likely caused by unhealthy eating behaviors in combination with little or no physical activity. On a global level, environmental factors, such as alcohol consumption, cigarette smoking, and the timing of onset of childhood obesity,^6^ drive the rise in the prevalence of obesity. There is strong evidence that body mass index (BMI) is determined by genetic factors, with its heritability ranging between 40% and 70%.^7^ In Sweden, a correlation for obesity onset based on BMI for monozygotic twins was observed to be 0.70 for males and 0.66 for females.^1^,^8^ Further studies on twins identified a 77% relationship between heritability and the prevalence of obesity based on BMI.^9^,^10^

The relationship between obesity and hypertension is well documented and has been observed in multiple studies and populations, in both sexes.^11^ Being obese increases an individual’s chances of developing high blood pressure 2- to 3-fold.^12^ In obese men, the mean systolic BP (SBP) and diastolic BP (DBP) values were 9 and 7 mm Hg higher. In obese women, these values were 11 and 6 mm Hg higher.^13^ Additionally, several lines of evidence indicate that regardless of race, age, or sex, high blood pressure is related to visceral or centrally located body fat rather than peripheral body fat.^14^–^16^ There are a variety of biologically active molecules that are produced from adipose cells. These include reactive oxygen species, pro-inflammatory and inflammatory molecules, angiogenic factors (such as vascular endothelial growth factor), homeostasis modulating compounds, and acute phase reaction proteins.^17^

The roots of obesity-linked hypertension can be traced to early childhood, 8–11 years, with the full effects being observed in adulthood.^18^ Hypertension is a major contributor to the development of heart disease, stroke, kidney failure, premature mortality, and disability. The 2010 report on the global burden of disease attributes 9.4 million deaths to hypertension.^19^ Moreover, obesity can be a major contributing factor for the resistance of hypertension to drug therapy. These patients blood pressure remains high despite being on three or more antihypertensive medications.^20^,^21^ Obesity-induced hypertension results from a complex interaction of genetic, behavioral, and environmental factors and the relationship between these factors and socio-economic status and lifestyle. Obesity-linked hypertension is accompanied by severe changes in cardiovascular and renal physiology, resulting from increases in plasma volume, tubular sodium retention, and heart rate, as well as an initial decrease in systemic vascular resistance. These physiological changes are in part due to and are accompanied by activation of both the sympathetic nervous system and the renin-angiotensin-aldosterone system (RAAS).^22^

Genome-wide association studies have identified single-nucleotide polymorphisms (SNPs) in the first intron of the *fat mass and obesity-associated* (*FTO*) gene also known as *alpha-ketoglutarate-dependent dioxygenase*. FTO is an RNA demethylase that regulates metabolic rate and body fat accumulation by controlling adipocyte differentiation into brown or white fat cells. These SNPs are strongly associated with BMI.^23^ The ability of the expression of genes to influence obesity or hypertension will be dependent on the expression of different isoforms of the protein encoded by these genes. This is because different isoforms may have widely different functions. Sometimes even acting against each other.^24^ L-type Cav1,2 calcium channel proteins are an example of alternatively spliced isoforms affecting blood pressure.^25^ This demonstrates the importance of genetic polymorphisms in contributing to obesity and hypertension. However, not all obese individuals are hypertensive, indicating that there is considerable variation in response to metabolic dysregulation. It is thought that interaction between obesity and genetic factors involved in the pathways that regulate blood pressure plays a significant role in the development of hypertension in obese individuals.

This implies that these genetic variants that contribute to the development of obesity can be identified and used to customize treatments to suit the patient, in order to prevent or delay the development of obesity-associated hypertension. Model organisms can provide useful information regarding the role played by genetic polymorphisms during developmental stages and how the influence of the polymorphisms during development can contribute to obesity and hypertension; these results can then be used in humans to help prevent age-specific onset of human obesity-related hypertension.

## Mechanisms to explain obesity-associated hypertension

**Figure 1:**
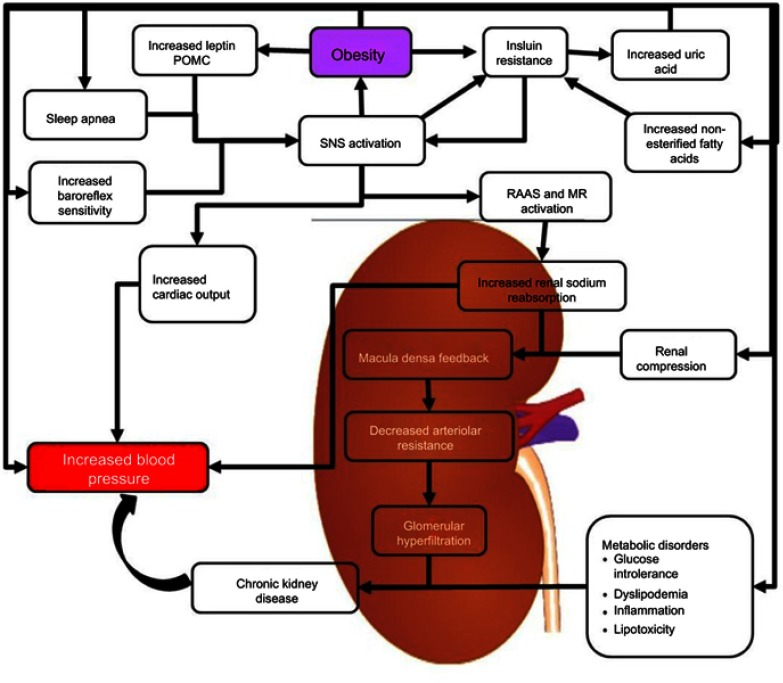
Mechanisms of obesity-related hypertension. The figure illustrates the different mechanisms by which obesity is able to contribute to increased blood pressure. **Abbreviations:** MR, Mineralocorticoid Receptors; POMC, Proopiomelanocortin; RAAS, Renin-Angiotensin-Aldosterone System; SNS, Sympathetic Nervous System.

The mechanisms that lead to obesity-induced hypertension are yet to be fully understood; however, it is likely that distinct hypertensive phenotypes with distinct genetic determinants are related to disease progression. There are several abnormalities in the central and peripheral vasculature that contribute to the development of high blood pressure in severely obese individuals. These include an increase in the activity of the sympathetic nervous system, insulin resistance, activation of the RAAS, and altered vascular function.^26^ The pathophysiological relationship between hypertension and obesity is interconnected with obesity initiating physiological responses that contribute to hypertension and hypertension triggering responses that could contribute to obesity as is shown in Figure 1.

### Sympathetic activation contributes to obesity-associated hypertension

An increase in the activity of the sympathetic nervous system (SNS) is a common feature of obesity, which is present in both animals and humans, and it plays an important role in hypertension progression.^27^ This obesity associated over activation of the SNS affects the heart, kidneys, and skeletal muscle tissues.^28^–^30^ Muscle sympathetic nerve activity (MSNA) is a good measure of SNS activity. Initially, it was unclear if there was a relationship between obesity and higher SNS activity, as this increase in SNS activity is not seen in all obese individuals. For example obese, hypertensive individuals have elevated cardiac SNS activity, while normotensive obese individuals have suppressed cardiac SNS.^31^ However, there is a great variation in the SNS activity of individuals of otherwise similarly obese individuals. When looking for a relationship between MSNA, obesity, and hypertension, it is seen that race, gender, environment, and genetics are all able to modify the impact of obesity-related neural activity has on arterial pressure. Differences in gender, for example, show MSNA is primarily related to BMI in men, but to blood pressure in women.^32^ The difficulty in observing a direct relationship between SNS activity and obesity was found to be due to the fact that SNS activity shows a stronger relationship to abdominal visceral fat than it does to total fat. This is clearly seen in populations such as the Native American Pima Indians. A large percentage of this population qualify as being obese. However, generally, they have low levels of sympathetic nerve activity in muscle. This may also be the reason that hypertension is uncommon in Pima Indians.^32^,^33^

It remains uncertain as to what causes the activation of SNS in obesity as there may be several factors involved. One such factor seems to be increased adrenergic activity.^34^ Dogs fed a high fat diet are prone to developing high blood pressure. Clonidine is a drug that acts as a central α_2_-receptor stimulator, resulting in reduced SNS activity. This is similar to α- and β-adrenergic blockers. When either clonidine or α- and β-adrenergic blockers are given to dogs fed a high fat diet, they helped to control the increased blood pressure normally observed in these dogs.^34^ This was also observed in humans where treatment of hypertensive individuals with a combination of α- and β-adrenergic blockers for 1 month resulted in a significantly greater reduction in blood pressure among obese individuals than in lean patients.^35^ Another factor that may contribute to SNS activation in obese individuals is insulin resistance; however, it has been proven that hyperinsulinemia does not promote hypertension.^36^,^37^ Another factor involved in SNS activation is the levels of leptin or other adipokines. Dysfunctional adipose tissue secretes excess leptin leading to altered levels of SNS activity.^27^ Leptin signaling results in the activation of *Signal Transducer and Activator of Transcription 3 (STAT3*). STAT3 acts downstream by downregulating the expression of endocabbanoids, which stimulate hunger. Disrupted STAT3 signaling leads to resistance to the anti-hunger effect of leptin, resulting in the consumption of excess food and possibly weight gain.^27^ Decreased leptin sensitivity in the ventromedial and dorsomedial hypothalamus involves signaling by PI3 kinase and melanocyte stimulating hormone and leads to enhanced SNS activity in the renal system.^27^,^38^ Other factors leading to increased SNS activity include renin-angiotensin activity and lifestyle. Visceral fat further contributes to the development of hypertension as it contributes to individuals developing obstructive sleep apnea. Sleep apnea is casually linked to hypertension and SNS activity due to intermittent hypoxia.^39^

### Insulin resistance

Obesity is defined by a state of impaired glucose tolerance, high levels of circulating insulin, and reduced sensitivity to the metabolic actions of insulin.^38^ This reduced sensitivity to the metabolic actions of insulin despite the high levels of insulin present is known as insulin resistance. While the increased levels of circulating insulin is known as hyperinsulinemia and has been proposed as an attempt to compensate for reduced insulin sensitivity. Hyperinsulinemia is also associated with impaired insulin-stimulated vasodilator action.^40^ High levels of centrally located abdominal or visceral fat surrounding the organs are associated with insulin resistance and high blood pressure.^27^,^41^ In borderline hypertensive individuals, insulin attains an acute sympathoexcitatory action. These individuals have a high muscle SNS activity and increased norepinephrine levels.^42^,^43^ In summary, resistance to insulin-stimulated glucose uptake and hyperinsulinemia is associated with obesity. Insulin sensitivity can be restored through weight loss. Therefore, insulin resistance may be considered as a link between obesity and hypertension.^2^,^3^,^41^

### Renin-angiotensin-aldosterone system (RAAS)

**Figure 2:**
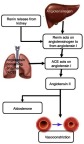
The RAAS is able to cause hypertension through the activity of the end products, angiotensin II and aldosterone. Angiotensin II acts on the blood vessels to cause vasoconstriction and increased blood pressure. Aldosterone leads to increased salt re-absorption in the kidneys, resulting in increased blood pressure. **Abbreviations:** ACE, Angiotensin-Converting Enzyme; RAAS, Renin-Angiotensin-Aldosterone System.

Obesity-induced hypertension is a major factor contributing to cardiovascular disease, Another factor contributing to both cardiovascular disease and hypertension due to obesity is the RAAS.^44^,^45^ The RAAS is responsible for maintaining normal blood pressure and fluid balance. It is activated in response to low blood pressure, a decrease in the volume of blood or filtrate flowing through the kidneys or a decrease in the serum levels of NaCl. The system is initiated by the juxtaglomerular cells in the kidneys converting pro-renin which is already present in the kidneys, into renin in response to low blood pressure. Renin cleaves a decapeptide from the angiotensinogen protein, which is secreted by the liver. The decapeptide is known as angiotensin I. Angiotensin I is then converted by the angiotensin-converting enzyme (ACE), found in the lungs, into angiotensin II by cleaving it into an octapeptide. Angiotensin II is a vasoconstrictor, narrowing blood vessels and raising blood pressure. Angiotensin II also stimulates Aldosterone secretion from the adrenal cortex. Aldosterone stimulates the increase in the reabsorption of NaCl and water in the kidneys, leading to increased blood pressure as a result of increased fluid volume^46^ (Figure 2).

The RAAS plays an important role in the development and maintenance of high blood pressure associated with obesity.^26^,^45^ Multiple reports indicate that the levels of active renin and angiotensin II are higher in the plasma of obese individuals, possibly because of higher SNS activity in the kidney.^47^,^48^ hypertension.^49^ Figure 1 depicts the multiple mechanisms that can activate the RAAS in obesity-induced hypertension. The development of hypertension because of insulin resistance such as in cardiometabolic syndrome (CMS) is partially caused by the interaction between RAAS and the sympathetic nervous system (SNS). In CMS, an increase in visceral adipose tissue, increased inflammation, and oxidative stress results in an increase in the activity of the renin-angiotensin system.^26^,^50^ In mice, the overexpression of angiotensin in adipose leads to an increase in blood pressure.^26^,^51^ This shows that Angiotensin I and II produced in adipose tissue has the potential to assist the growth and expansion of adipocyte tissue and has systematic effects on the regulation of blood pressure.^26^

Targeting the RAAS in hypertensive obese patients can result in a decrease in hypertension as well as in the incidences of associated metabolic disorders such as type 2 diabetes. Drugs that can be used to target the RAAS include ACEs or angiotensin II receptor blockers.^52^ The contribution of the RAAS to hypertension in obese individuals is further complicated by the fact that adipose tissue expresses all components of the RAAS. Activation of the RAAS in adipose tissue is associated with high blood pressure in both animal models of visceral obesity^53^,^54^ and obese hypertensive patients.^55^ The presence of angiotensinogen in adipose tissue may have further implications in obesity-linked hypertension, as Ang II plays a major role in the growth and differentiation of adipocytes in rodents.^56^

### Alteration of vascular function

**Figure 3:**
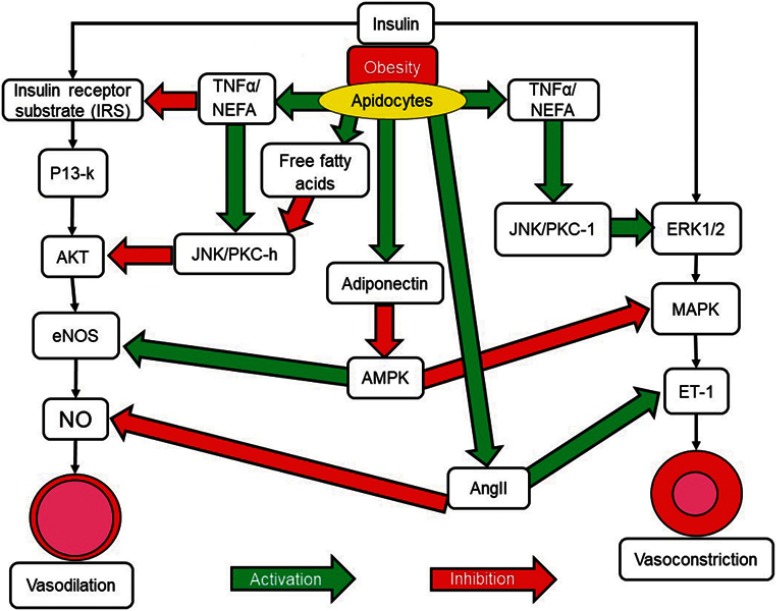
Insulin and the alteration of vascular function. Insulin acts through two separate pathways in vascular cells to promote either vasodilation or vasoconstriction. The IRS/P13K/Akt pathway promotes vasodilation. The MAPK pathway promotes vasoconstriction. Obesity leads to the expression of molecules that can inhibit either pathway, leading to an imbalance in vasodilator and vasoconstrictor actions. Green arrows represent stimulation of pathways or stimulatory signals. Red arrows represent inhibition of pathways or inhibitory signalling. **Abbreviations:** Akt, Protein kinase B; AngII, Angiotensin II; ET-1, Endothelin 1; eNOS, endothelial Nitric Oxide Synthase; ERK ½, Extracellular signal–Regulated Kinase 1/2; IRS, Insulin Receptor Substrate; JNK, c-Jun N-terminal Kinases; MAPK, Mitogen-Activated Protein Kinases; NEFA, Non-Esterified Fatty Acids; NO, Nitric Oxide; PI3-k, Phosphoinositide 3-kinase; PKC-II, Protein Kinase CII; TNF-α, Tumor Necrosis Factor alpha.

There is increasing evidence concerning the important role played by vascular endothelial dysfunction in the pathogenesis of hypertension. Obesity is a known contributing factor to endothelial dysfunction. Insulin signaling pathways stimulate inflammation and lead to aberrant excessive and uncontrolled endothelial growth resulting in endothelial dysfunction and hypertension (Figure 3). Endothelial nitric oxide derived from vascular endothelium provides protection against inflammation and platelet aggregation while at the same time also promoting vasodilation. The activation of insulin-dependent phosphoinositide 3-kinase causes phosphorylation of endothelial nitric oxide synthase and an increase in production of nitric oxide while at the same time hyperinsulinemia leads to an increase in vasoconstrictor endothelin-1 levels. This is due to activation of the mitogen-activated protein kinase pathway (MAPK) by endothelin-1. This results in a disturbance in the balance between vasodilation and vasoconstriction in the vascular endothelium^57^,^58^(Figure 3).

MAPK pathways also regulate the production of adhesion molecules by the endothelial cells. These adhesion molecules include vascular cell adhesion molecule-1, intercellular adhesion molecule-1, and E-selectin and promote the adhesion of monocytes to the vascular wall.^17^,^57^,^59^ This may contribute to obesity being a major risk factor for carotid atherosclerosis. The progression of atherosclerosis causes large arteries to become stiffer. Furthermore, aortic pulse wave rises in obese patients due to stiffening of the aorta and aberrant restructuring of other smaller arteries, Arterial stiffness and pulse wave velocity are both enhanced in obesity-associated hypertension.^17^,^59^

### Renal and adrenal mechanisms

The kidneys and adrenal glands play an important central role in the regulation of blood pressure. Obese individuals and subjects with metabolic syndrome tend to be relatively salt sensitive.^60^,^61^ One of the ways in which the kidney can regulate blood pressure is through altered sodium retention or excretion. Increased retention of sodium results in increased blood pressure.^26^ Increased sympathetic nerve signaling to the kidneys may lead to increased sodium reabsorption in the kidneys. This was seen in obese dogs with high blood pressure, where renal denervation resulted in lower levels of sodium reabsorption and retention, which in turn decreased blood pressure in these dogs. Sodium excretion in the urine can also be impaired by high amounts of fat surrounding the kidneys as well as increased visceral fat. Other factors associated with the kidneys ability to regulate blood pressure include BMI, waist circumference, and insulin resistance.^20^,^61^,^62^

Sodium retention is also associated with plasma aldosterone levels. Aldosterone is a steroid hormone produced by the adrenal gland. It is the main mineralocorticoid hormone in the body and is essential for the control of sodium retention in the kidney. Aldosterone levels in African American individuals are associated with hypertension. These hypertensive individuals also display high plasma aldosterone concentrations. The effect of obesity on aldosterone levels is partly due to the ability of adipokines to stimulate aldosterone production.^45^ Once again dogs fed on a high-fat diet were used as a model to study obesity-induced hypertension. In this case, they were used to study the role of aldosterone on obesity-induced hypertension. Sodium retention and the associated development of hypertension in obese dogs were prevented by treating the dogs with the mineralocorticoid antagonist, eplerenone. Eplerenone blocked aldosterone binding to mineralocorticoid receptors (MR) in the kidney and showed the effect that selective aldosterone receptor antagonists can have on the development of hypertension.^44^

High concentrations of cortisone may also bind to mineralocorticoid receptors leading to an increase in arterial pressure in a similar way, but to a lesser degree, than aldosterone.^49^ Rodent models lose weight following the removal of the adrenal glands (adrenalectomy); however, treatment of these individuals with glucocorticoid replacement therapy leads to hypercoticosteronemia and weight gain. Obese individuals have high levels of 11b-hydroxysteroid dehydrogenase-1 (11β-HSD1) expressed in their adipose tissues.^38^ This enzyme is responsible for reducing cortisone to the active hormone cortisol and is known as an NADPH-dependent cortisone reductase. It is activated in many tissues, including adipose tissue. The conversion of cortisone to cortisol is higher in visceral fat than it is in subcutaneous fat.^63^ The mutant mouse strain aP2-HSD1 has the expression of 11β-HSD1 under the control of the enhancer-promoter region of the gene that codes for the adipocyte fatty acid–binding protein (aP2). These transgenic mice can be induced to overexpress 11β-HSD1 in fat cells, leading to visceral obesity with insulin resistance, dyslipidemia, and hypertension. Studies using these mice suggest that the glucocorticoid activity in adipose tissue leads to the activation of the RAS pathway, which then leads to increased salt-sensitivity and a resulting increase in hypertension in obese mice.^38^,^64^ Despite this, the contribution of cortisone to the development of hypertension in obese individuals is unclear as the levels of circulating cortisol vary in obese individuals.^49^

## Genetics of obesity and hypertension in humans

The development of obesity and hypertension in individuals involves behavioral, environmental, and genetic factors. Evidence of a genetic component is strongly implicated as playing a significant role in the risk of becoming obese.^65^ The study of twins has commonly been used as a model to assess the genetic component of a given trait. Monozygotic twins that are genetically identical can be compared to non-identical dizygotic twins that only share 50% of their genetic component. In monozygotic twins, the genetic component that contributes to the development of obesity ranges from 70% to 90%. The genetic component is less in dizygotic twins (35–45%).^66^ Hypertension is also driven by a genetic component in patients with monogenic traits; however, it can also be influenced by environmental factors. These can include salt intake and diuretic use. Recent developments in sequencing platforms and technology have allowed for the rapid identification of genes and genetic mutations associated with obesity and hypertension. Next-generation sequencing in combination with traditional Sanger sequencing has identified more than 50 SNPs associated with hypertension or high blood pressure. These SNPs affect genes that are involved in signaling pathways that affect processes such as renal function, and natriuresis.^56^ A high-throughput next-generation study demonstrated that hypertension-associated genetic loci were rare; however, those variants that were identified were found to have larger effects on blood pressure than common variants. Gene variants encoding monogenic forms of hypertension were also identified, indicating that there is a significant proportion of variability in the genetic causes of blood pressure.^65^,^67^,^68^

Genome-wide association studies (GWASs) are able to detect if gene variants are associated with certain traits such as body mass index and obesity, with large GWAS meta-analysis revealing a number of genes that may influence an individual’s susceptibility to obesity.^63^,^69^ One such study identified 97 gene variants that were related to BMI, with 56 of these being novel. Susceptibility loci were also identified in both adults and children. One of the genes that has been identified to have variants associated with obesity was the *fat mass and obesity-associated gene (FTO)*.^70^ Certain variants of both the *FTO* and *GNDPA 2* genes are associated with obesity in the Chinese population. Multiple other genes have variants that may predispose individuals to obesity.^71^–^74^ Many of these genes are highly expressed in the hypothalamus and other areas within the central nervous system, implicating these genes as playing a role in energy homeostasis and dietary intake.^70^ Table 1 lists genes that are implicated in obesity.

**Table 1:** Genes implicated in obesity and hypertension

Gene	Protein encoded by the gene	Loci	Reference
*FTO*	Fat mass and obesity associated	16q12.2	23
*RBM4A*	RNA-binding factor a	11p14.1	79
*LEP*	Leptin	7q32.1	134
*TCF7L2*	Transcription factor 7-like 2	10q25.2	73
*TMEM18*	Transmembrane protein 18	10q25.5	135
*SH2B1*	SH2B containing domain 2	16p11.2	135
*MC4R*	Melanortin 4 receptor	18q21.32	134
*KCTD15*	Potassium channel tetramerization domain containing 15		136
*BDNF*	Brain-derived neurotrophic factor	11p14.1	134
*SEC16B*	SEC16 homolog B	1q25.2	137
*PCSK1*	Proprotein convertase subtilisin/kexin type 1	5q15	134

## Alternative splicing

The splicing of precursor messenger RNA (pre-mRNA) occurs post-translationally and results in different isoforms of a protein, encoded by a single gene, being expressed. Alternative splicing (AS) allows for the generation of a greater diversity of proteins without increasing the number of genes. Alternative splicing generates these different isoforms by incorporating and excluding different combinations of exons, resulting in different mature mRNA molecules. The resulting protein isoforms may have related, distinct, or even opposing biological properties.^62^ The tightly regulated process of AS provides the cell with the ability to adapt rapidly to intra and extracellular stimuli. AS occurs in the vast majority of all human genes and results in an average of six protein isoforms per gene. This is why the 22,000 predicted protein-coding genes of the human genome can give rise to >200,000 observed protein isoforms.^63^ The prevalence of AS in an organism is directly related to that organism’s complexity. This implies that AS has evolved to play an important role in virtually all biological processes.^75^,^76^ The proper regulation of AS is required for the normal progression of these biological processes and aberrant AS is associated with disease.^77^,^78^

### Rbm4a

**Figure 4:**
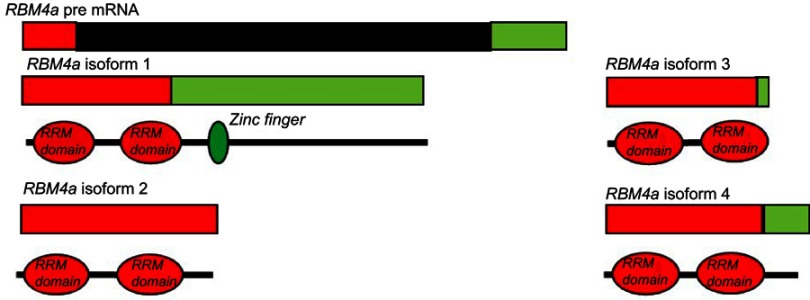
Alternative splicing of RBM4α. The pre-mRNA of *Rbm4a* contains 2 exons. The first exon (red) encodes two RNA recognition motifs (RRM) and is present in all isoforms. The second exon (green) contains internal splice sites resulting in isoforms 2, 3, and 4 having versions of this exon. In the full length isoform 1 this exon codes for a zinc finger. **Abbreviation:** *RBMA, RNA-Binding factor A gene*.

The *RNA-binding factor A (Rbm4a)* gene is located on chromosome 11 at position q13.2. This gene has multiple functions in cellular processes such as the regulation of both AS and translation. RBM4α modulates 5-splice site and exon selection, thereby regulating AS. It is responsible for regulating the splicing of genes such as *L-type Cav 1,2*. The different isoforms of which play different roles in developmentally regulating blood pressure in the heart.^25^ Not only does RBM4A regulate AS, but it is alternatively spliced to give rise to four isoforms (Figure 4). These isoforms differ due to the presence of a zinc finger domain in the longest full-length isoform. In muscle tissue, RBM4A functions as a modulator of alternative splicing and promotes the differentiation of muscle cells by regulating the alternative splicing of the heterogeneous nuclear ribonucleoprotein, *Polypyrimidine tract-binding protein 1* (*PTBP1*) by activating exon skipping during muscle cell differentiation. PTBP1 is a splicing factor and it controls the splicing of *alpha tropomyosin* in muscle cells. RBM4A antagonizes the splicing modulation role of PTBP1.^79^ The downregulation of the expression of PIB1 and PTB-2 is required for apidogenic development.^79^

Knockdown studies in mice showed that RBM4A is associated with proper pancreas development and therefore the expression of insulin. RBM4A also controls insulin receptor splicing switches.^80^ In addition to this, RBM4A controls the splicing switches of many genes involved in metabolism and energy production, such as *pyruvate kinase*, contributing to changes in mitochondrial energy production in differentiating cells.^81^ RBM4A controls the translation of the *Period circadian 1 protein (PER1)* mRNA. PER1 plays an important role in controlling circadian rhythm and cell cycle progression in cells. Under stress conditions, it facilitates the initiation of the translation of specific target mRNAs by binding to ribosome entry sequences on these mRNAs. This results in the recruitment of the translation initiation factor EIF4A1 and the initiation of Internal Ribosome Entry Site IRES-dependent translation.^79^,^82^ It also binds to CU-rich responsive elements within the 3ʹUTR of mRNAs, suppressing Cap-dependent translation.

### FTO

The FTO protein is a member of the AlkB family of non-heme Fe (II)/dioxygenases. *FTO* was the first gene identified to contribute to non-syndromic human obesity.^23^ It has been demonstrated using FTO overexpressing or knockout mouse models that *FTO* is associated with abnormal adipose tissues and body mass. Characteristics that suggest a role for *FTO* in adipogenesis and energy homeostasis.^23^ The *FTO* locus was found to be associated with BP. Individuals homozygous for the rs9930333 SNP in the *FTO* locus had a greater BMI by 1.8 kg/m^2^ and a higher blood pressure, 3.6 mm Hg higher than normal.^83^ FTO is able to de-methylate various methylated nucleic acid (both RNA and DNA) bases,^84^–^86^ with N6-Methyladenosine (m6A) being an abundant modification in mRNA that promotes mRNA translation and stability. The ability of FTO to regulate RNA stability through modification with m6A was found to be related to obesity, with m6A modification being more common during adipogenesis. Analysis of mRNA populations containing m6A modifications in combination with transcriptome analyses demonstrated that m6A modification was more common in exons that were adjacent to intronic 5ʹ/3ʹ splice sites on either side of spliced exons. It was also observed that the sites of m6A modification overlapped with sequences recognized by the splicing enhancer, Serine and Arginine Rich Splicing Factor 2 (SRSF2). Since FTO functions to regulate the RNA-binding ability of SRSF2, as well as m6A demethylation, FTO performs the role of an RNA splicing regulator, a function which explains its vital role during adipogenesis.^23^

**Figure 5:**
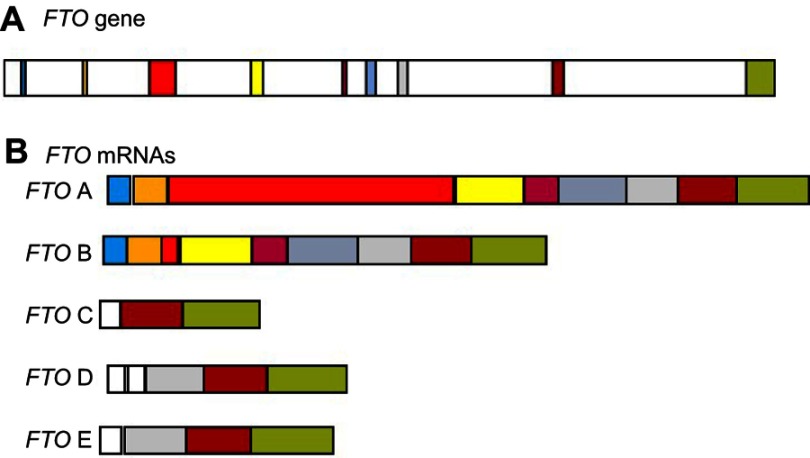
Alternative splicing of the FTO mRNA. (**A**) Exon structure of the pre-mRNA **(B)** This pre-mRNA is spliced to give multiple isoforms. *FT*O *A* contains all of the exons. *FTO* B contains a truncated exon 3 due to internal splice sites. The identity of each exon and its position in each isoform is indicated by different colour blocks. **Abbreviation:** *FTO, Fat mass and obesity-associated gene*.

The full-length *FTO* gene consists of nine exons (Figure 5) with the catalytic AlkB domain being encoded by exons 3–5. Therefore, only isoform A has a fully functional AlkB domain. Like humans, chicken *FTO* mRNA is alternatively spliced to give rise to multiple isoforms (cFTO1-cFTO4). Only isoforms encoded by *cFTO1* and *cFTO4* retain demethylase activity. In the chicken, the expression of these various isoforms is associated with glucose metabolism, body weight, fatness, and body composition. Feeding chickens a high-glucose or high fat diet results in higher expression levels of cFTO1 in all tissues except in the pituitary.^87^

### Leptin receptor

Another possible link between obesity and the development of hypertension is hyperleptinemia. Leptin is a hormone produced by adipose tissue in direct proportion to adipose tissue mass.^59^ The effects of Leptin (LEP) on appetite are due to its ability to increase the metabolic rate and influence thermogenesis.^88^,^89^ Leptin also plays an important role in metabolism and disease in other organs such as the kidney and heart. It also plays a role in the stimulation of the sympathetic nervous system and systematic vasculature.^17^,^90^,^91^ Leptin has been associated with blood pressure control, effects on appetite and energy utilization as well as nutrient absorption.^71^ Hypertensive patients have high plasma levels of leptin. In obese patients, there is an interaction between increased renal sympathetic tone and high leptin levels.^71^ Leptin deficiency leads to obesity in both animal models^89^ and humans.^88^

**Figure 6:**
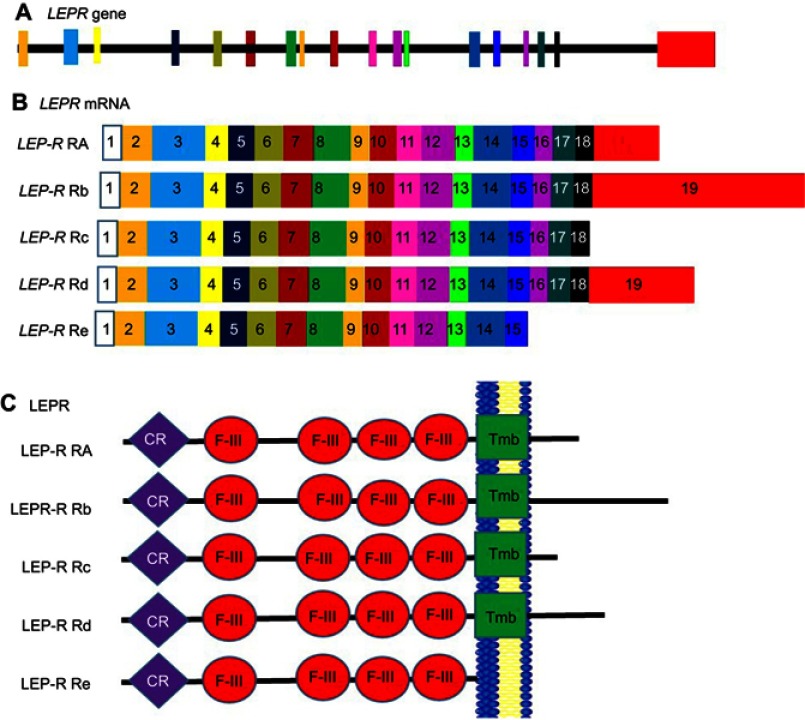
Alternate splicing of *LepR*-mRNA. Schematic figure representing leptin receptor splice variants. (**A**) *Lep-r* gene showing the exon structure, (**B**) *Lep-r* splice variants, showing the different isoforms. Exons are represented by numbered boxes. The different colours of the boxes corresponds to the position of each exon on the *Lep-r* gene in panel **A**. (**C**) Resulting LEP-R isoforms. Exon 17 encodes the transmembrane domain (green). The cytokine receptor domains (purple) and the fibronectin 3 domains (red) are found in all isoforms. **Abbreviations:** CR, Cytokine Receptor domain; F-III, Fibronectin type 3 domain; Lep-R, Leptin receptor; Tmb, Transmembrane domain.

Variations in the 5ʹ-region of *leptin* are known to be associated with an increase in body mass Index (BMI) and obesity.^92^ Polymorphisms in the *leptin* gene are also associated with obesity. One such polymorphism known as *C532T* involves a base pair change at the 3ʹ-UTR and is associated with increased blood pressure.^93^ The leptin receptor (Lep-R) exists as multiple isoforms that arise due to alternative splicing (Figure 6). It exists as several isoforms. These isoforms can be grouped into three classes, namely, the long, short, and secretory leptin receptor classes. The N termini of these isoforms are the same, giving rise to similar extracellular domains in all Lep-R isoforms.^94^ The only isoform that is fully active in terms of signal transduction is the long isoform LPR-Rb. This isoform is involved in energy homeostasis and immunity.^94^ In the C57BL/KsJ- db/db mutant mouse model of type 2 diabetes, it is expressed in low levels resulting in these mice displaying an early obesity phenotype.^95^ The shorter isoforms (Lep-Ra, Lep-Rc, and Lep-Rd) function to internalize and degrade Leptin. Increased expression of these isoforms leads to a decrease in Leptin signaling.^96^ Leptin binds to the short form receptors and is able to cross the blood–brain barrier and enter an area of the hypothalamus known as the arcuate nucleus. This hypothalamic region helps to regulate appetite through the transmission of neuropeptides, which control appetite, to peripheral tissues. The results of leptin activity on the hypothalamic neuronal system include reduced food intake, increased thermogenesis, and higher energy expenditure through stimulation of sympathetic neural activity.^12^,^97^ The shortest secretory isoform Lep-Re lacks the intracellular, cytoplasmic, and transmembrane domains. It is suspected that this isoform regulates leptin levels by delaying leptin clearance. It may also regulate leptin activity by competing with membrane receptors for ligand binding. A decrease in the expression of this shorter isoform leads to an increase in obesity.^94^

### TCF7L2

Transcription factor 7-like 2 (TCF7L2) is a transcription factor that contains a high mobility group (HMG) domain. These domains are involved with DNA binding and protein–protein interactions. TCF7L2 plays a role in the Wnt signaling pathway and is associated with blood glucose homeostasis. Mutations in *TCF7L*2 increase the chances of the individual developing type 2 diabetes.^73^ This gene has multiple different isoforms encoded by several transcript variants some of these variants increasing the risk of developing type 2 diabetes. TCF7L2 variants are one of the best biomarkers for the diagnosis of type 2 diabetes. However, in some non-diabetic obese–hypertensive patients, certain variants are associated with increased BMI and high fasting glucose levels.^98^ It is likely that alternative splicing in conjunction with SNPs in the *TCF7L2* gene leads to impaired TCF7L2 function. Different splice variants and expression patterns are found in different tissues.^99^ Human *TCF7L2* consists of 18 exons and is characterized by alternative splicing of 3ʹ end exons 12, 13, 13a, and 13b. These alternate exons contain novel stop codons, leading to truncated isoforms because of the termination of transcription. The full exon 14 is required to code for the c terminus protein-binding domain (CTBP), which inhibits the Wnt signaling pathway. Exon 13 encodes a c-Clamp (CRARF) domain, which activates the Wnt signaling pathway. Since the Wnt signaling pathway is important in insulin signaling, it is also involved in the development of type 2 diabetes.^98^ Overexpression of the full length TCF7L2 has a protective effect on β-cell survival. Overexpression of the short variant lacking exons 12–13 and 13a led to impaired insulin secretion and β-cell apoptosis.^99^,^100^

### Insulin receptor

The insulin receptor is activated through the binding of insulin, IGF-I and IGF-II. It is a tyrosine kinase receptor and exists in two isoforms in humans. The smaller isoform, IR-A, is normally expressed in the developing fetus. It lacks exon 11, which codes for 12 amino acids at the c terminus. The larger IRB isoform is normally expressed in adults. The isoforms have different binding affinities for (IGF)-2 and proinsulin, with an increase in the expression of IR-A being associated with diabetes and hyperinsulinemia.^101^ Weight loss also leads to an increase in the expression of IR-B.^102^ The insulin receptor was one of the first genes to be associated with hypertension. Different polymorphisms in different exons are associated with hypertension. These polymorphisms are found in exons 9, 8, and 2. Individuals possessing these forms of insulin receptor show a family history of hypertension.^103^ These different isoforms of receptor have different affinities for insulin and result in different insulin signaling intensities. Insulin signaling can increase angiotensinogen levels.^103^,^105^

### LMNA

**Figure 7:**
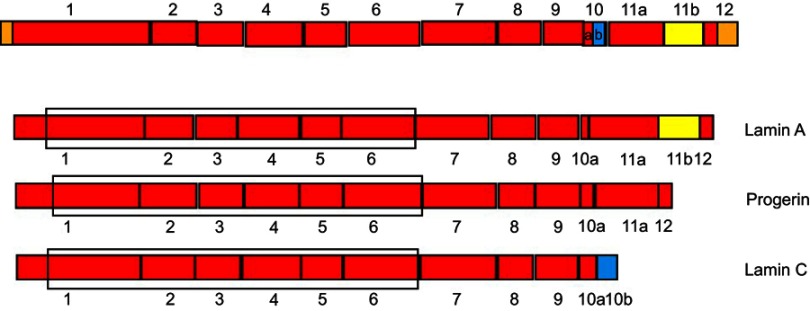
Schematic figure representing LMNA splice variants. (**A**) Lamin pre-mRNA. The longest splice variant Lamin A contains all exons except 10b (green). Progerin contains exon 11b not 11a (yellow) and 10b. Only the shortest isoform Lamin C contains exon 10b, where it codes for the C terminus of the protein. The box marks the region coding for the central dimerization domain. **Abbreviations:** NCoR, Nuclear Receptor Corepressor; RIDs, Receptor Interaction Domains.

Lamin is a fibrous protein that acts as a structural component of the nuclear membrane and regulates transcription in the nucleus. Mutations in *LMNA* are associated with lipodystrophy, hyperinsulinemia, dyslipidemia, diabetes, and hypertension.^105^^,^^106^ *Prelamin* (*LMNA*) mRNA contains 12 exons (Figure 7[Fig F0008]) and is alternatively spliced to produce three isoforms, lamin A, progerin, and lamin C. Lamin A and C differ due to splicing of exon 10, while progerin differs from Lamin A due to alternate splicing of exon 11 (Figure 7).^101^ This results in progerin being 50 amino acids shorter than lamin A at the C terminal. Obese and type 2 diabetic patients have elevated expression levels of lamin c in their subcutaneous adipose tissue.^107^ Overexpression of Lamin C or a knockdown of progerin and Lamin A results in an obese phenotype with decreased energy metabolism and mitochondrial activity. Progerin is antagonistic to Lamin C signaling and overexpression of Lamin C results in higher energy metabolism and the inability to produce and maintain healthy fat tissue.^108^

### Lipin-1

**Figure 8 F0008:**
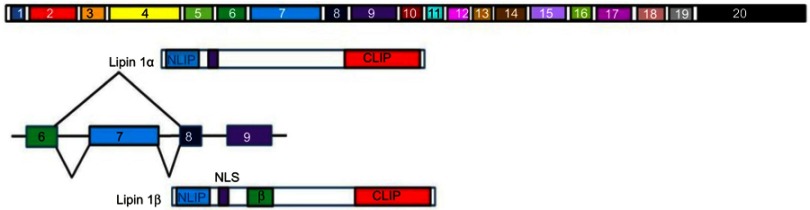
Representation of Lipin alternative splicing. Lipin-1α lacks exon 7 (light blue), which codes for the 33 amino acid long β domain (lime green) seen in Lipin-1β. The NH2 terminal (NLIP) and COOH terminal (CLIP) domains are also shown. These domains are highly conserved across the Lipin family. **Abbreviation:** LMNA, Lamin.

Lipin-1 is a phosphatidate phosphatase (PAP) enzyme that functions to convert phosphatidate to diacylglycerol and is therefore required for triacylglycerol synthesis. Lipin 1 also acts as an inducible transcriptional coactivator and in combination with the peroxisome proliferator-activated receptor α (PPARα) and PPARγ coactivator 1α (PGC-1α), it serves to regulate fatty acid metabolism. Lipin-1 is therefore required for adipocyte differentiation and lipid metabolism and influences fat mass and energy balance in adipose tissue and skeletal muscle.^109^ Lipin 1 plays a role in blood pressure regulation, with some alleles containing SNPs being associated with low blood pressure. This effect was greater in men.^110^ Lipin-1 is alternatively spliced to give rise to seven isoforms. Two of these isoforms Lipin-1A and Lipin-1B are expressed at different stages of adipocyte differentiation. Lipin 1A is expressed in the early stages of pre-adipocyte differentiation while Lipin 1B is expressed at increasing levels as differentiation progresses. These two isoforms differ due to the inclusion of exon 7 in Lipin-1B (Figure 8[Fig F0008]). Lipin-1A induces the expression of transcription factors such as peroxisome proliferator-activated receptor gamma (PPARγ) and CCAAT-enhancer-binding protein-alpha (C/EBPα), both of which promote differentiation. Lipin-1B induces lipogenesis.^111^ The relative expression of these two isoforms is controlled by the RNA splicing factor SFRS10 and Sirtuin 1 (SIRT1), which regulates the metabolic response to caloric restriction. Lipin-1 is a splicing target of SFRS10 and a decrease in the levels of SFRS10 results in an increase in the expression of Lipin-1B.^102^ SIRT1 expression is inhibited by ethanol, which results in reduced SFRS10 expression and therefore an increase in Lipin-1B expression accompanied by a decrease in Lipin-1A expression.^5^,^102^

## Therapies to treat obesity-linked hypertension

Currently, there are no specific guidelines to treat hypertension associated with obesity-related hypertension and no adequate large-scale drug trial have been performed specifically targeting this problem. However, the vast majority of patients with hypertension are also overweight or obese. Any treatment to lower blood pressure should not contribute to weight gain. Choices of an appropriate treatment should be based on the mechanisms of obesity-induced hypertension, as well as clinical experience with these patients. Other important factors to consider would be patient comorbidities, side effects, and the frequency and cost of treatment.^112^,^113^

### Treatment of hypertension in obese patients

Antagonizing the RAAS is a common drug target for lowering blood pressure. Targeting this system has the added advantage that the RAAS contributes to obesity. These drugs have an advantage over other commonly used drugs used to treat hypertension such as thiazide diuretics and β‐blockers, as the use of any of these classes of drugs can result in insulin resistance.^114^

Metabolically neutral agents such as angiotensin-converting enzyme inhibitors (ACEIs), angiotensin receptor blockers (ARBs), direct renin inhibitors, and mineralocorticoid receptor antagonists are the first-line choice of drugs for obesity-related hypertension.^43^ ACEs and ARBs have the added advantage of contributing to the control of obesity by improving glucose tolerance, increasing insulin levels and decreasing the levels of visceral fat﻿. Therefore, these drugs would also decrease the risk of patients developing type 2 diabetes mellitus.^115^ There are currently two types of drugs on the market for the treatment of obesity.^109^ The first, Orlistat, inhibits lipase and therefore reduces the amount of fat absorbed by the intestine.^116^–^119^ The second drug, sibutramine, is an anorectic or appetite suppressant.^120^ However, both of these drugs are expensive and have side effects such as increased blood pressure, dry mouth, constipation, headache, and insomnia.^121^–^124^ This has led many to explore the use of naturally occurring compounds, mostly isolated from plants, to treat obesity.^123^

These are mainly complex mixtures of multiple components, each with its own chemical and pharmacological features. The combination of multiple phytochemicals may have synergistic activity that increases their bioavailability and their action on multiple molecular targets, which outweighs that of any single constituent.^60^,^125^,^126^ Natural compounds that have anti-obesity effects act in various ways. These include decreasing lipid absorption, altering the intake and expenditure of energy, increasing lipolysis, decreasing lipogenesis, and decreasing pre-adipocyte differentiation and proliferation.^127^ Traditional healers of many cultures rely on plants to provide therapeutic and medicinal compounds. These plants are used to treat multiple ailments and have led to plants being a center of research for obesity and other diseases. Plant extracts and compounds isolated from plants are known to have the ability to manage obesity due to their effects on metabolism and fat oxidation.^29^,^128^ In addition to this, some medicinal plants have shown potential as treatments for obesity-related ailments such as hypertension and diabetes.^126^,^128^ Various types of tea have shown the ability to decrease energy intake from the gastrointestinal tract. These include green, oolong, and black tea, which are known to act on pancreatic lipase. Table 2 shows some of the medicinal plants that can regulate or reduce the onset of obesity.

**Table 2:** Medicinal plants considered as treatments for obesity

Plant species or common name	Patient description	Effect of plant extract on patient	Reference
*Camellia sinensis*	Obese with metabolic syndrome	Decrease in BW and BMI	138
*Opuntia ficusindica*	Obese, pre diabetic	Decreased blood glucose	139
*Satiereai sativus L*	Mildly overweight women	BW reduction	140
*Nigella sativa*	Obese male	Reduced BW, WC, and SBP	141
Pomegranate seed oil	Hyperlipidemia	Decreased TG and TG; HDL cholesterol ratio	142
Northern berries	Women	Changes in calorie intake, WC and increased plasma adiponectin levels	143
Oolong tea	Diet induced obesity	Decrease in BW and subcutaneous fat	144
*Irvingia gabonensis*	Overweight to obese	Decreased BW, WC, and body fat	145
*Pistachio*	All	Weight loss decreased BMI	146

**Abbreviations:** BW, Body weight; BMI, Body mass index; HDL, High-density lipoprotein; SBP, Systolic blood pressure; TG, Triglyceride; WC, Waist circumference.

### Regulation of splice site variants in diseased patients

Proper regulation of alternative splicing relies on splicing machinery that can accurately and precisely identify the splice sites for intron removal and exon joining. Even a small error, such as single nucleotide addition or deletion, will shift the reading frame, leading to alteration of protein function. The splicing machinery responsible for achieving this accuracy is the spliceosome. The spliceosome consists of 5 snRNAs packaged with approximately 80 proteins.^129^ The availability of tissue-specific splice factors may regulate naturally occurring processes in the cell. This ability of the cell to regulate alternative splicing may be affected by mutations changing the location of splice sites. This, in turn, may lead to skipping or addition of exons, resulting in protein function change causing disease. This process of dysregulated or incorrect alternative splicing is known to occur in those genes that play a role in obesity and hypertension.^99^,^129^,^130^

Missense mutations have been predicted to cause diseases by contributing to 1.6% of all splicing errors and approximately 7% of the exonic variants may alter splicing which includes cryptic splicing.^126^ Misplicing is an attractive drug target to treat diseases that are caused by splicing errors due to mutations. These therapies include the use of small molecule modifiers and antisense oligonucleotides.^130^ Metformin is a drug that was developed for the treatment of type 2 diabetes. It reduces hepatic glucose production while increasing the rate at which glucose is utilized. It also alters the composition of gut microbiota and increases the expression of glucagon-like peptide 1 (GLP-1). GLP-1 reduces blood sugar levels by increasing the expression of insulin. Metformin is able to modify alternative mRNA splicing by activating cAMPK and downregulating the RNA-binding protein 3 (RBMA).^131^ Another splicing factor that can be targeted by drugs is the serine and arginine-rich splicing factor 1 (SRSF1), also known as Alternate Splicing Factor 1 (ASF-1). This splicing factor promotes exon skipping and any drug that inhibit or regulate the activity of SRSF1 will change the relative amounts of different isoforms. A small indole derivative, IDC16, is able to target SRSF1. IDC16 was used as the basis to design the small molecule inhibitor of SRFS1, ABX300. ABX300 acts against the development of obesity by acting on SRSF1 to change the levels of LMNA isoforms. ABX300 treatment was able to correct misplicing caused by high fat diets and led to an alteration in the metabolic rate by increasing the expression of genes that prevent obesity and increased fat loss.^132^

Antisense oligonucleotides can act as splicing switches by binding to enhancer or silencing sequences within the target mRNA and influence the binding of splicing factors to these sequences.

These splice switching oligonucleotides (SSO) have been used in trials to treat multiple ailments and seem to have few side effects for the patient.^133^

## Conclusion

Alternative splicing of the mRNA coding for genes that play a role in metabolism gives rise to protein isoforms that play important and sometimes conflicting roles in metabolic pathways. These metabolic pathways can influence an individual’s weight gain. Alternatively, these genes can control the development of adipocytes, resulting in obesity. Obesity is a major contributor to the development of hypertension. Regulation of alternative splicing or abnormal mRNA splicing can therefore be a major contributing factor to the development of obesity and consequently obesity-linked hypertension. The full effect of SNPs in obesity-linked loci is unknown, but SNPs may alter the splicing of genes associated with obesity and obesity-linked hypertension. This will then result in changes in the composition of the proteasome. One of the more important pieces of data that is missing from our knowledge regarding the role of alternative splicing in obesity-linked hypertension is the contribution of environmental factors to changes in the population of splice variants. Further characterization of splicing variants may allow these splice variants to be used as diagnostic or prognostic markers for metabolic and hypertensive disorders. They may also serve as lead targets for the development of drugs. The current strategies of using small molecules or antisense oligonucleotides to regulate and correct aberrant splicing are both promising avenues of investigation. One of the major sources for small molecules that may act on splicing is plants and plant-based compounds.
